# Evaluating Nanoparticle Breakthrough during Drinking Water Treatment

**DOI:** 10.1289/ehp.1306574

**Published:** 2013-08-09

**Authors:** Talia E. Abbott Chalew, Gaurav S. Ajmani, Haiou Huang, Kellogg J. Schwab

**Affiliations:** Department of Environmental Health Sciences, Johns Hopkins Bloomberg School of Public Health, Baltimore, Maryland, USA

## Abstract

Background: Use of engineered nanoparticles (NPs) in consumer products is resulting in NPs in drinking water sources. Subsequent NP breakthrough into treated drinking water is a potential exposure route and human health threat.

Objectives: In this study we investigated the breakthrough of common NPs—silver (Ag), titanium dioxide (TiO_2_), and zinc oxide (ZnO)—into finished drinking water following conventional and advanced treatment.

Methods: NPs were spiked into five experimental waters: groundwater, surface water, synthetic freshwater, synthetic freshwater containing natural organic matter, and tertiary wastewater effluent. Bench-scale coagulation/flocculation/sedimentation simulated conventional treatment, and microfiltration (MF) and ultrafiltration (UF) simulated advanced treatment. We monitored breakthrough of NPs into treated water by turbidity removal and inductively coupled plasma–mass spectrometry (ICP-MS).

Results: Conventional treatment resulted in 2–20%, 3–8%, and 48–99% of Ag, TiO_2_, and ZnO NPs, respectively, or their dissolved ions remaining in finished water. Breakthrough following MF was 1–45% for Ag, 0–44% for TiO_2_, and 36–83% for ZnO. With UF, NP breakthrough was 0–2%, 0–4%, and 2–96% for Ag, TiO_2_, and ZnO, respectively. Variability was dependent on NP stability, with less breakthrough of aggregated NPs compared with stable NPs and dissolved NP ions.

Conclusions: Although a majority of aggregated or stable NPs were removed by simulated conventional and advanced treatment, NP metals were detectable in finished water. As environmental NP concentrations increase, we need to consider NPs as emerging drinking water contaminants and determine appropriate drinking water treatment processes to fully remove NPs in order to reduce their potential harmful health outcomes.

Citation: Abbott Chalew TE, Ajmani GS, Huang H, Schwab KJ. 2013. Evaluating nanoparticle breakthrough during drinking water treatment. Environ Health Perspect 121:1161–1166; http://dx.doi.org/10.1289/ehp.1306574

## Introduction

Engineered nanoparticles (NPs) are currently used in > 1,200 commercially available consumer products, including personal care products, food storage containers, cleaning supplies, bandages, clothing, and washing machines (Reijnders 2006). These products release NPs into the domestic waste stream during use, cleaning, and disposal, leading to NPs in surface waters (Benn and Westerhoff 2008; Blaser et al. 2008; Mueller and Nowack 2008). Estimated concentrations of NPs in U.S. surface waters range up to 10 μg/L silver (Ag), 24.5 μg/L titanium dioxide (TiO_2_), and 74 μg/L zinc oxide (ZnO) NPs (Blaser et al. 2008; Gottschalk et al. 2009; Mueller and Nowack 2008). Concentrations in surface water are anticipated to increase over time with greater use and disposal of NP-containing products (Klaine et al. 2008).

Because of increasing NP concentrations in surface waters, it is important to consider the ultimate fate of NPs. When suspended in the water column, NPs are likely to affect aquatic organisms (Handy et al. 2008) and be present in surface waters used as source water for drinking water treatment plants. Environmental fate and transport of NPs are largely related to NP dissolution (Elzey and Grassian 2010) and aggregation of single NPs into larger agglomerates, which are more likely to settle out of suspension (Petosa et al. 2010). The extent of aggregation, final NP size, and interaction with natural organic matter (NOM) will impact the efficiency of NP removal during drinking water treatment (Hyung and Kim 2009; Zhang et al. 2008).

Water treatment is one of the main strategies to prevent the ingestion of harmful contaminants, including NPs, from drinking water (Hyung and Kim 2009). Conventional drinking water treatment typically involves coagulation, flocculation, and sedimentation. During coagulation, a chemical, such as alum, is added to destabilize dissolved particles [U.S. Environmental Protection Agency (EPA) 1999]. During flocculation, destabilized particles aggregate into larger flocs, which can then be removed by gravity sedimentation (Crittenden et al. 2005). To monitor effective drinking water treatment for NOM removal, the U.S. EPA has set guidelines for the required total organic carbon (TOC) removal by coagulation based on initial TOC and alkalinity (see Supplemental Material, Table S1) (U.S. EPA 1999). Water treatment plants use turbidity and TOC as surrogate measures for NOM and contaminant removal.

In addition to conventional treatment, the use of low pressure membrane (LPM) filtration as an advanced water treatment technology has increased in prevalence over the past two decades (Huang et al. 2009). Unlike conventional treatment, LPM filtration relies on physical sieving to remove particulate contaminants (Crittenden et al. 2005). Therefore, the pore size of membranes employed in LPM filtration is expected to affect the removal of NPs in water.

Removal of NPs through drinking water treatment is not well understood. TiO_2_ and ZnO NPs spiked into buffered ultrapure and tap water achieved > 60% removal using alum coagulation and sedimentation (Zhang et al. 2008). Using carbon fullerene NPs (nC_60_) spiked into synthetic freshwater, NP removal by simulated conventional treatment was correlated with NOM concentration (Hyung and Kim 2009). However, these studies did not test NP removal in natural waters with complex chemistries that can affect NP aggregation, dissolution, and removal. In addition, in these studies, removal of metal oxide NPs was determined using graphite furnace atomic absorption spectroscopy with sensitivities in the milligram per liter range (Zhang et al. 2008). Use of more sensitive instrumentation, such as inductively coupled plasma–mass spectrometry (ICP-MS) with sensitivity in the nanogram per liter range, is necessary to accurately assess removal of NPs and metal ions from drinking water. The lack of accurate detection of NPs in finished drinking water has limited our understanding of NP exposure via this route.

The ingestion of NPs via drinking water may pose a potential direct human health threat or an indirect risk due to release of metal ions from the NPs. Exposure to metal NPs or metal NP ions via ingestion can result in adverse effects including kidney damage, increased blood pressure, gastrointestinal inflammation, neurological damage, and cancer (Kavcar et al. 2009; U.S. EPA 2013; Vahter et al. 2002). Cell uptake, cytotoxicity, and DNA damage in the Caco-2 human intestinal cell line have been reported after *in vitro* NP exposure (Abbott Chalew and Schwab 2013; Gaiser et al. 2012; Gerloff et al. 2009, 2012; Koeneman et al. 2010).

Exposure to NPs via the ingestion of drinking water, tested using *in vivo* animal studies, has revealed adverse effects. Rats and mice that ingested metal NPs had increased metal concentrations in their liver, kidneys, brain, and blood compared with controls (Kim et al. 2008; Park et al. 2010; Wang et al. 2007). Cha et al. (2008) and Park et al. (2010) reported histological evidence of inflammation, as well as increased liver enzymes related to necrosis and inflammation, in rats and mice in response to Ag and ZnO NPs in drinking water. The ingestion of metal NPs has also been reported to lead to DNA damage (Sharma et al. 2012; Trouiller et al. 2009). The consequences of increased metal burdens, DNA damage, and liver toxicity are not fully understood. However, these studies indicate that the ingestion of NPs can lead to NPs or metal ions in systemic circulation with potentially adverse consequences.

The objectives of the present study were to investigate the removal of NPs during conventional and advanced water treatment, determine the effects of NP and water properties on the removal process, and investigate the magnitude of NPs and released ions not removed by the treatment processes (“breakthrough”). The experiments were conducted using Ag, TiO_2_, and ZnO NPs commonly present in consumer products that are also likely to be present in water. These NPs were chosen to replicate previous studies and to represent potential environmental fate of NPs: stabilization as individual particles (Ag NPs), aggregation to larger aggregates (TiO_2_ NPs), and dissolution into metal ions (Ag and ZnO NPs). We investigated removal of NPs from groundwater, surface water, synthetic water with and without NOM, and tertiary wastewater effluent. We used jar tests to simulate conventional treatment (coagulation/flocculation/sedimentation) and LPM filtration to simulate advanced water treatment. The removal of NPs was evaluated by traditional water quality parameters such as turbidity removal, TOC removal, and ultraviolet (UV)/visible light absorbance. In addition, we used ICP-MS for advanced quantification to further elucidate removal.

## Materials and Methods

*Nanoparticles.* Ag, TiO_2_ (Aeroxide P25), and ZnO NPs were purchased from Sky Spring Nanomaterials Inc. (Houston, TX), Evonik Degussa (Pasippany, NJ), and NanoAmor (Houston, TX), respectively. The size and shape of NPs were determined by transmission electron microscopy (Phillips EM 420; Phillips, Amsterdam, the Netherlands). All particles were spherical or semispherical with average NP sizes of 83.6 nm, 33.7 nm, and 35.6 nm for Ag, TiO_2_, and ZnO NPs, respectively (see Supplemental Material, Figure S1).

We prepared stock NP suspensions by weighing NPs on a Mettler Toledo microbalance (0.1 μg sensitivity; Mettler-Toledo, Columbia, MD), transferring weighed NPs into 15-mL polypropylene tubes, and adding ultrapure water (Millipore, Billerica, MA) to achieve 100 mg/L. The suspensions were vortexed for 10 sec and pulse sonicated at 20 kHz for 4 min with 0.5-sec pulses using a 550 Sonic Dismembrator (Fisher Scientific, Pittsburgh, PA). Suspensions were stored at room temperature for up to 1 week and resonicated before each experiment.

*Water characterization.* We selected five test waters for NP removal experiments: a Maryland groundwater source (GW) currently used for drinking water, a suburban surface water source (SW) from central Maryland, synthetic freshwater (SFW) with and without NOM, and tertiary wastewater effluent (WWeff) from Maryland. The SFW was prepared with 50 mg/L sodium bicarbonate, 30 mg/L calcium sulfate, 30 mg/L magnesium sulfate, and 2 mg/L potassium chloride (all Optima grade reagents; Fisher Scientific). The NOM source was water collected from the Great Dismal Swamp National Wildlife Refuge in southeastern Virginia (Huang and O’Melia 2008); NOM was diluted into SFW to a final concentration of 5 mg carbon/L (SFW_NOM; for NOM characterization, see Supplemental Material, Table S2). Water samples were prefiltered using 1.2-μm glass fiber filters (Whatman GF/C) and stored at 4°C. Waters were fully characterized (see Supplemental Material, Table S3).

*Characterization of nanoparticles in water.* Using stock suspensions, NPs were diluted 1:10 (10 mg/L) into test waters and characterized for size using dynamic light scattering (Zetasizer ZS90; Malvern Instruments, Westborough, MA) (Table 1). We used this 10-mg/L concentration of NPs to remain within the sensitivity range of the analytical instruments. The *z*-average values are reported as the mean of three samples of each NP in each water type with four measurements of 10 runs of 10 sec each. Measurements were rejected if the count rate was < 100 kilocounts and the polydispersal index was > 0.7.

**Table 1:** Size of NPs (z‑average ± SD) in test waters measured using dynamic light scattering.

Water type	Ag NPs (nm)	TiO_2_ NPs (nm)	ZnO NPs (nm)
Groundwater	223.4 ± 81.1	271.6 ± 16.6	292.6 ± 16.8
Surface water	390.3 ± 90.5	1,147 ± 234	353.6 ± 50.1
Synthetic freshwater	248.1 ± 68.4	3,338 ± 984	7,021 ± 5,066
Synthetic freshwater with NOM	199.0 ± 38.6	248.1 ± 8.03	321.1 ± 89.0
Tertiary wastewater effluent	421.3 ± 82.3	751.3 ± 102	354.8 ± 50.9
Values represent three samples measured four times each per water type.			

*Metals analysis.* We determined NP removal from water by measuring total metal content (a surrogate for NP) by ICP-MS (Agilent, Santa Clara, CA). A 300-μL aliquot of sample was combined with 700 μL concentrated nitric acid (HNO_3_; Optima grade; Fisher Scientific) in a 7-mL Teflon vessel and microwaved (Mars Express; CEM, Matthews, NC) using a program that raised the temperature to 165°C over 20 min, 175°C over an additional 7 min, and held at 175°C for 30 min. Each sample was diluted with ultrapure water to 2% HNO_3_. HCl (Optima grade; Fisher Scientific) was added to reach 0.5%, and 50 ppb of indium and scandium (Agilent, Santa Clara, CA) were added as an internal standard. Thus, all samples had a final concentration of 0.5% HCl and 2% HNO_3_.

We converted output from the ICP-MS into a mass per unit volume value, which was blank corrected using ultrapure water as a method blank. Unspiked waters were also digested to obtain background metal contamination levels in the waters, which we used to background correct for each experimental water. Untreated NPs (1 mg/L in ultrapure water), which served as the reference material, were prepared for each experiment and processed along with the samples. The limits of detection (LOD) were 3.95, 0.35, and 0.16 μg/L for Ag, Ti, and Zn, respectively. For statistical analysis, total metal concentrations below the LOD were substituted with a value of one-half the LOD.

*Jar tests.* We used a programmable jar tester (Phipps & Bird, Richmond, VA) to determine the optimal coagulant dose of alum. Test waters were spiked with 1 mg/L NPs from stock suspensions and mixed overnight with a magnetic stir bar at 60 rpm. Reagent-grade aluminum potassium sulfate [KAl(SO_4_)_2_ × 12H_2_O; i.e., potassium alum] was dissolved in ultrapure water for a 2.8-g Al/L stock solution, which was stored at 4°C.

To determine the optimal alum dose, we added a range of alum doses to 250-mL glass beakers holding 150 mL spiked test water that was being stirred at 25 rpm at room temperature. The water was mixed rapidly at 100 rpm for 2 min, mixed slowly at 25 rpm for 30 min, and allowed to settle for 60 min. Turbidity and pH of the supernatants were measured immediately using a 2100N turbidimeter (HACH, Loveland, CO) and an AR20 pH/conductivity meter (Fisher Scientific), respectively. Supernatants were stored in polypropylene centrifuge tubes at 4°C until TOC and ICP-MS analysis. Jar tests were conducted in triplicate for each NP in each water type.

We considered the optimal alum dose as the minimum dose required to effectively remove both turbidity and TOC; this method is similar to that employed in full-scale drinking water treatment. If the optimal dose for turbidity and TOC were not the same, we selected the higher dose. Jar tests were also conducted to determine the optimal alum dose for SFW_NOM without NPs (*n* = 3).

*Membrane filtration.* Concurrently with the jar test experiments, two membrane filtration experiments were conducted using the same batch of test water. NP aliquots of 1 mg/L NPs in experimental waters were filtered through a polyvinyldine fluoride (PVDF) flat-sheet membrane (Millex; Millipore) with a nominal pore size of 0.45 μm for microfiltration (MF) or a ceramic flat-sheet membrane (Whatman, Anotop 25) with a nominal pore size of 0.02 μm for ultrafiltration (UF).

Ultrapure water was pumped through both types of membranes at 1 mL/min until a stable UV signal was reached. Spiked test waters were then pumped through the membranes at 1 mL/min for 120 min. UV absorbance was monitored throughout filtration by UV spectral scans at 254 nm, 320 nm, and 370 nm for Ag, TiO_2_, and ZnO NPs, respectively. We prepared a composite sample of filtrate for each NP per water type per filtration method by collecting filtrate for 15 sec at 1, 5, 30, 60, 90, and 120 min. Samples were stored at 4°C until ICP-MS analysis.

We also prepared composite samples for each water type without NPs, as described above for spiked samples. These composite samples were analyzed by ICP-MS and used to background correct the results of spiked water experiments. All experiments were conducted in triplicate for each NP in each water type.

*Statistical analyses.* Statistical analyses were conducted using SigmaPlot 11 (Systat Software, San Jose, CA) and STATA 11 (StataCorp LP, College Station, TX). Comparison of two means was conducted using Student’s *t*-test, and comparison of multiple means was conducted using one-way analysis of variance (ANOVA). We used the Holm–Sidak method, Dunn’s one-way ANOVA on rank, or the Tukey test to determine significance among multiple pair-wise comparisons.

## Results

*Nanoparticle removal by alum coagulation.* We determined the optimal alum dose for each NP and test water combination (Table 2). The optimal doses determined by turbidity or TOC removal were not significantly different for each experimental condition (test water and NP). Optimal alum doses were significantly different for ZnO NPs compared with both TiO_2_ and Ag NPs in SFW (*p* < 0.05) and for ZnO NPs compared with Ag NPs (*p* < 0.01) in WWeff (Table 2) measured for turbidity. All of the optimal alum doses met the goals for percent NOM removal according to the Enhanced Coagulation Rule (see Supplemental Material, Table S1), which sets guidelines for percent NOM removal based on the initial TOC and alkalinity (U.S. EPA 1999).

**Table 2:** Optimal alum dose (mg Al/L) in each water type for each NP as determined by turbidity and TOC removal.

Water type	Ag NPs		TiO_2_ NPs		ZnO NPs	
	Turbidity	TOC	Turbidity	TOC	Turbidity	TOC
GW	4.36 ± 0.33	3.98 ± 1.14	4.55 ± 0.57	3.55 ± 0.28	3.98 ± 0.57	3.22 ± 1.19
SW	3.41 ± 0.57	3.79 ± 0.85	3.22 ± 0.33	3.27 ± 0.55	2.75 ± 0.17	2.84 ± 0.57
SFW	2.62 ± 0.42	2.84 ± 0.52	2.66 ± 0.68	2.78 ± 0.97	1.21 ± 0.14*	1.42 ± 0.57*
SFW_NOM	3.32 ± 0.16	3.70 ± 0.74	3.05 ± 0.22	3.41 ± 1.54	3.36 ± 0.37	3.84 ± 0.55
WWeff	10.2 ± 0.97**	10.64 ± 1.31	9.40 ± 0.33	9.95 ± 1.42	8.36 ± 0.33**	9.10 ± 0.57
Abbreviations: GW, groundwater; NOM, natural organic matter; SFW, synthetic freshwater; SFW_NOM, synthetic freshwater with NOM; SW, surface water; TOC, total organic carbon; WWeff, wastewater effluent. Values represent three experiments per NP in each water type. *p < 0.05 compared with other NPs. ** *p *< 0.01, between ZnO NPs and Ag NPs.						

The optimal alum dose for SFW_NOM without NPs, based on turbidity removal, was 3.13 ± 0.3 mg Al/L (see Supplemental Material, Figure S2). This dose was not statistically different from the optimal dose for SFW_NOM containing any type of NPs (Table 2; see also Supplemental Material, Figure S2).

**Figure 1:**
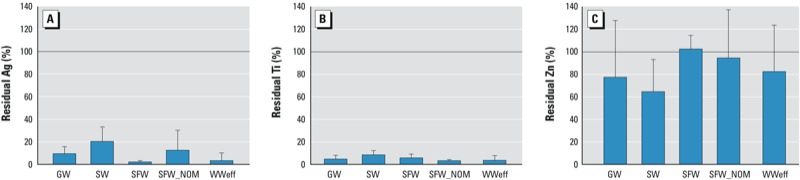
Percent NP breakthrough (residual; mean ± SD) of Ag (*A*), Ti (*B*), and Zn (*C*) in ground­water (GW), suburban stream (SW), synthetic fresh­water (SFW), SFW with NOM (SFW_NOM), and tertiary waste­water effluent (WWeff) after simulated conventional treatment, as determined by ICP-MS (*n* = 3 per NP per water type). The reference line indicates 100% breakthrough.

We analyzed the supernatant of the optimal dose for total metals by ICP-MS and estimated NP breakthrough (Figure 1). Breakthrough of NPs was lowest for Ag and TiO_2_, with approximately 20% and < 10% breakthrough, respectively, for all water types. ZnO had the highest breakthrough, with > 60% in most waters and complete breakthrough in SFW (Figure 1). We observed no statistically significant difference in breakthrough within each NP by water type. However, ZnO breakthrough was statistically different from both Ag and TiO_2_ for the same water type (*p* < 0.05; Figure 1). Breakthrough was confirmed, as metals were detected in most finished water samples, with ranges of 0–305 μg/L for Ag, 0–465 μg/L for Ti, and 344–3,200 μg/L for Zn [see Supplemental Material, Table S4, “Coagulation/Flocculation/Sedimentation” (CFS)].

**Figure 2:**
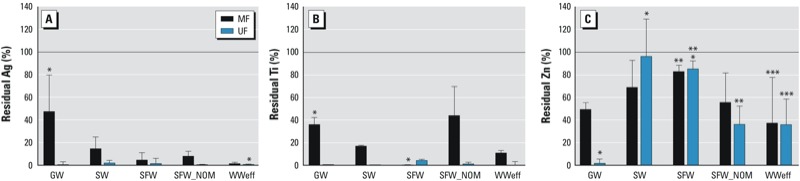
Percent NP breakthrough (residual; mean ± SD) of Ag (*A*), Ti (*B*), and Zn (*C*) in finished waters after micro­filtration (MF) or ultra­filtration (UF) of ground­water (GW), suburban stream (SW), synthetic fresh­water (SFW), SFW with NOM (SFW_NOM), and tertiary waste­water effluent (WWeff), as determined by ICP-MS (*n* = 3 per NP per water type). The reference line indicates 100% breakthrough. *p < 0.05 for differences between waters within NP type and filtration method. **p < 0.05, and ***p < 0.01 for Zn compared with both Ag and Ti within water type and filtration method.

*Removal by membrane filtration.* The composite MF and UF samples were analyzed by UV absorbance and ICP-MS to estimate NP removal (Figure 2). UV absorbance was measured throughout the membrane removal experiments, and higher UV absorbance indicates that more NPs passed through the membrane into the filtered water. The UV absorbance results were similar for all NPs in all waters, with greater UV absorbance in MF filtrates than in UF filtrates (see Supplemental Material, Figure S3). However, the UV signals from both MF and UF were low and not statistically different from the test water containing no NPs, indicating a low breakthrough of NPs. The results from ICP-MS indicate that NPs that broke through MF varied by both NP type and water type. After MF, Ag, Ti, and Zn were detected at concentrations up to 743, 1,330, and 2,261 μg/L, respectively. After UF, Ag, Ti, and Zn NPs were detected at concentrations up to 44, 158, and 3,202 μg/L, respectively (see Supplemental Material, Table S4).

Overall, less breakthrough resulted from UF than from MF (Figure 2). UF resulted in < 5% breakthrough for Ag and TiO_2_ from all waters and for ZnO from GW (see Supplemental Material, Table S5). Breakthroughs from MF were < 20% for Ag NPs from all waters except GW and for TiO_2_ NPs from all waters except GW and SFW_NOM. However, breakthroughs for ZnO NPs were significantly higher than those for Ag and TiO_2_ NPs from MF and UF in all waters (see Supplemental Material, Table S5).

## Discussion

*NP breakthrough during conventional treatment.* NPs can be removed through coagulation if they are enmeshed by the coagulate floc as it sediments out of the water, in a process called sweep floc (Crittenden et al. 2005). Alternatively, coagulants added to the water may affect NP stability by producing positively charged hydrolytic species that neutralize negative surface charges on NPs (Westerhoff et al. 2008), resulting in greater NP aggregation because electrostatic repulsion is mitigated (Petosa et al. 2010).

A key operational parameter for coagulation is coagulant dose. For conventional treatment, water characteristics drive the optimal coagulant dose (O’Melia et al. 1999; Shin et al. 2008). In the present study, optimal doses for GW, SW, and SFW_NOM were similar regardless of the NP type (Table 2), and the optimal alum dose was not statistically significantly different for SFW_NOM with or without NPs (Table 2; see also Supplemental Material, Figure S2). These results confirm that NOM was the main driver for optimal coagulant doses and not the addition of the NPs to the water (even at a high particle concentration of 1 mg/L). Because of the relative abundance of NOM to NPs in our experiments, hydrolytic aluminum species formed from potassium alum will preferentially react with the free NOM (Hyung and Kim 2009; O’Melia et al. 1999) instead of NPs in water.

The low expected environmental concentrations of NPs in surface waters are less likely to affect the optimal coagulant dose in water treatment plants. The optimal alum dose for drinking water treatment is typically determined by evaluating removal of turbidity or TOC following jar tests (Crittenden et al. 2005; U.S. EPA 1999). In our experiments, use of traditional measurement techniques to determine optimal dose resulted in finished waters that met the U.S. EPA coagulation guidelines (U.S. EPA 1999). However, the optimal coagulant dose for TOC removal required by the U.S. EPA guidelines was not sufficient for removal of the NPs because we found detectable levels of Ag, Ti, and Zn in all the finished waters at levels above those detected in the test water containing no NPs (see Supplemental Material, Table S3). These metals may be NPs, ions released from NP surfaces, or fully dissolved NPs. Increased metal concentrations in finished waters, whether NPs or ions, is a concern for human health and for water treatment.

*NP breakthrough during membrane filtration.* Filtration by LPM is increasingly being used for drinking water treatment and potable water reuse (wastewater reuse) (Guo et al. 2010; Huang et al. 2009). In the present study, we assessed MF using a 0.45-μm PVDF membrane, which is at the upper pore size limit of membranes employed by full-scale membrane filtration plants (Van Der Bruggen et al. 2003). We investigated UF using a 0.02-μm ceramic membrane, which was smaller than the NP primary particle size and had pore sizes that were uniformly 20 nm (Van Der Bruggen et al. 2003).

**Figure 3:**
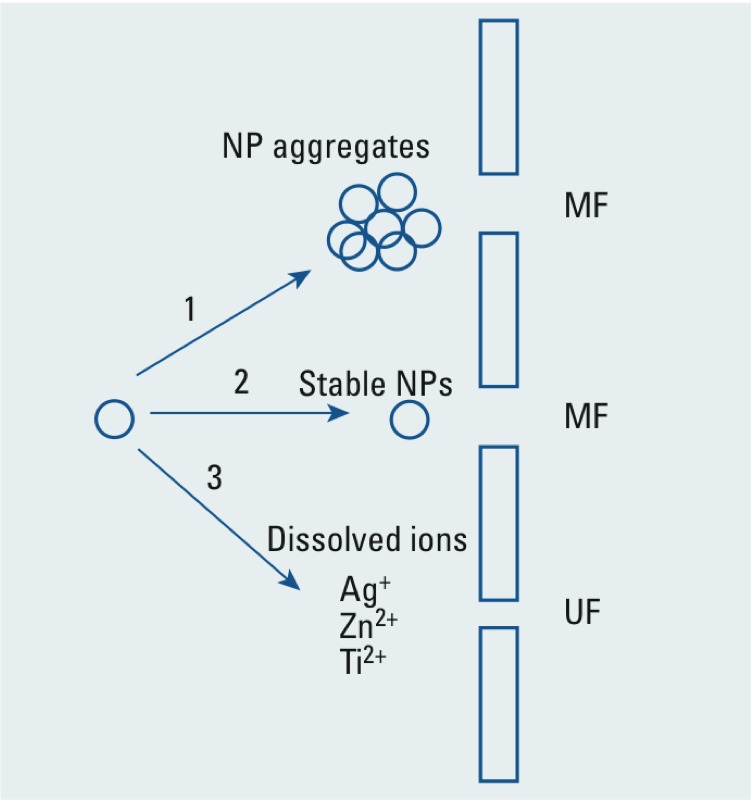
Mechanisms of NP stability and removal by membrane filtration. The NP can 1) aggregate, 2) remain as a single NP, or 3) dissolve into ions. Stable NPs or ions would pass through micro­filtra­tion (MF), but not aggregates. Only ions would pass through the ultrafiltration (UF) membrane.

Removal of compounds and contaminants by LPM filtration is predominantly based on physical sieving effects (Crittenden et al. 2005; Westerhoff et al. 2008). Therefore, removal of NPs by membrane filtration depends on membrane pore size, NP size, and NP stability—either aggregation or dissolution. We outlined three potential outcomes for NPs in source water used for drinking water treatment: NPs aggregating with other NPs or NOM in the water, NPs remaining as primary particles, or NPs dissolving into ions (Figure 3). In experimental waters containing NOM (all except SFW), the NPs aggregated to sizes ranging from 199–421 nm, 248–1147 nm, and 292–355 nm for Ag, TiO_2_, and ZnO NPs respectively (Table 1). The extent of aggregation was influenced by the characteristics of the experimental water (Table 1). Most aggregates will be larger than the membrane pore sizes utilized in LPM filtration and thus will not pass into finished drinking water. Our results confirm that MF was most effective for larger NP aggregates. Small NP aggregates or stabilized NPs, such as Ag and TiO_2_ in GW and SFW_NOM (Table 1), could break through the membrane.

Investigation into NP dissolution was facilitated by UF, because the pore size was smaller than the primary particles. Therefore, any metals detected in the UF filtrate should be ions. UF has been used by other researchers to distinguish soluble species of metals (Ussher et al. 2010). At the pH and ZnO NP concentration used in most of the experiments, Zn(II) was the predominant species, indicating ZnO NP dissolution into zinc ions (Stumm and Morgan 1996). Dissolution of ZnO NPs was confirmed by similar NP removals between MF and UF: Only dissolved ions could break through UF (Figure 3), and UF removal of ZnO NPs was greatest from the GW source water, which had the highest initial pH. However, ZnO NP dissolution was incomplete because there was some ZnO removal in all waters by MF and some ZnO NP aggregates measured by dynamic light scattering, which indicated that some ZnO NPs remained as NPs (Table 1).

Consistent removal of stabilized NPs will occur only if membrane pore sizes are smaller than NPs, such as the UF membrane used in these experiments. However, dissolved ions released by ZnO NPs passed through UF membranes and thus would require filtration with even tighter membranes or the use of other treatment technologies not commonly used in large-scale drinking water treatment, such as ion exchange. For potable water reuse, reverse osmosis has frequently been used after LPM filtration or conventional treatment, and should serve as an effective barrier for dissolved metal ions.

*Implications for drinking water treatment and public health.* NPs are increasing in both production and the environment; therefore, it is important to understand human exposures to NPs, especially as the literature on the adverse effects of NPs increases. Drinking water treatment provides a barrier to contaminant exposure via ingestion of drinking water. However, our results suggest the occurrence of NP breakthrough into finished water after coagulation/flocculation/sedimentation, as well as membrane filtration. Although experimental NP concentrations were higher than expected environmental concentrations, the removal efficiencies are not expected to change significantly with changing concentration.

As illustrated in Figure 3, NP aggregate size is an important parameter for NP removal. Larger aggregates will be removed by settling floc during conventional treatment and by physical separation during advanced membrane filtration.

Investigation into NP size in source waters can inform the choice of treatment technology, including membrane pore size. Enhanced NP removal by LPM filtration must be balanced with increased energy costs and greater membrane fouling that is likely to occur when NOM is present in source water. Moving forward, it may be necessary to combine treatments in sequence for optimal removal of NPs (Moon et al. 2009; Zhang et al. 2008).

We found that although use of traditional drinking water quality parameters, such as TOC and turbidity removal, resulted in finished water that met U.S. EPA guidelines for coagulation, the water still contained detectable metals (see Supplemental Material, Table S5). More sensitive detection methods and instrumentation, such as ICP-MS, were necessary to accurately determine NP removal and measure residual metals in finished waters. These new methods are more expensive and time consuming and thus may be impractical for water utilities to use to continuously monitor quality of finished water. To protect public health by monitoring NP removal during drinking water treatment, new, simpler, online detection methods for NPs in water will be necessary.

Although the health effects of ingesting NPs, especially at low concentrations, are unknown, we believe that it is important to apply the precautionary principle and begin to consider NPs as emerging drinking water contaminants. In our experiments, the concentrations of NPs detected in finished waters were below the concentrations reported to damage intestinal cells *in vitro* (Abbott Chalew and Schwab 2013; Koeneman et al. 2010) but high enough to cause adverse effects to aquatic organisms (Bowman et al. 2012; Gaiser et al. 2012). However, NPs and metal ions released from NPs will only increase over time with greater production and use of NP-containing consumer products, leading to a potentially greater health risk. Despite high removals from conventional and advanced treatment, we detected metals, possibly NPs, in finished waters. Until the health effects of NP ingestion are better understood, we need to develop appropriate removal processes for both the NPs and the released ions in order to protect public health into the future.

## Conclusions

As NP-containing products increase, there is a greater likelihood that NPs will contaminate drinking water resources. Using NPs spiked into synthetic and natural waters, our estimations indicated NP breakthrough following conventional and advanced drinking water treatment. Simulated conventional treatment resulted in 10–20% NP breakthrough; membrane filtration, especially UF, was more effective than conventional treatment for NP removal. Despite high removals, finished waters contained detectable metal concentrations that may pose hazards to human health. NP removal by both treatment processes was affected by NP stability including aggregation and dissolution. When NPs dissolve, as we observed with ZnO NPs, other treatment processes may be required. NPs should be considered an emerging drinking water contaminant, and their removal during drinking water treatment should be monitored to protect public health.

## Supplemental Material

(1.1 MB) PDF
