# Supplementary material for: Evaluating Nanoparticle Breakthrough during Drinking Water Treatment
Source: Environ Health Perspect. 2013 Aug 9;121(10):1161–6. doi: 10.1289/ehp.1306574 (PMC3801469; doi:10.1289/ehp.1306574)
Supplement: (1.1 MB) PDF [file ehp.1306574.s001.pdf]

Supplemental Material

Evaluating Nanoparticle Breakthrough during Drinking Water Treatment

Talia E. Abbott Chalew, Gaurav S. Ajmani, Haiou Huang, and Kellogg J. Schwab

Table of Contents

Table S1. EPA guidelines for total organic carbon removal by coagulation.

Table S2. NOM fractionation of experimental waters containing NOM measured with size exclusion chromatography and a TOC analyzer.

Table S3. Characterization of the test waters used for experiments.

Table S4. Range of metals detected in finished drinking water samples.

Table S5. Comparison of NP removal (%) by conventional and advanced treatment determined by mass by ICP-MS (n=3 experiments for each NP in each water type ± standard deviation).

Figure S1. Transmission electron micrographs of a) silver, b) TiO_2, and c) ZnO NPs in ultra-pure water.

Figure S2. Optimal alum dose for turbidity removal from synthetic freshwater with NOM with and without 1mg/L spiked Ag, TiO_2, and ZnO NPs (n=3 for each NP in each water as well as SFW_NOM without any NPs, average and standard deviation).

Figure S3. Sample curve of UV absorbance of 1 mg/L TiO_2 NPs in SFW_NOM following micro- and ultra- membrane filtration.

Table S1. EPA guidelines for total organic carbon removal by coagulation.

Water type	Total organic carbon (TOC) (mg/L)	Alkalinity (mg $CaCO_3$/L)	Guideline: percent TOC removal required	Final TOC (mg/L) to meet guideline
Groundwater	0.83	184	15	0.7055
Surface Water	2.41	76.5	25	1.8075
Synthetic Freshwater	0.41	56	35	0.2665
Sythetic freshwater with NOM	5.37	62	35	3.4905
Wastewater effluent	11.43	175	30	8.001

Table S2. NOM fractionation of experimental waters containing NOM measured with size exclusion chromatography and a TOC analyzer. NOM fractions include large molecular weight compounds (colloidal material), medium molecular weight compounds (humic material), and smaller molecular weight compounds.

NOM Fraction	Large molecular weight (colloidal) (%)	Medium molecular weight (humic) (%)	Small molecular weight (%)	Other (%)
Groundwater	0	23.4	75.8	0.8
Surface water	7.8	59.2	32.7	0.3
Synthetic freshwater with NOM	7.5	82	7.8	2.7
Wastewater effluent	3.7	96.3	0	0

Table S3. Characterization of the test waters used for experiments.

Parameter	Ground-water	Surface Water	Synthetic Freshwater	Synthetic Freshwater with NOM	Tertiary Wastewater Effluent
pH[a]	8.27	7.54	7.55	7.54	7.80
Total organic carbon (mg/L)[b]	0.83	2.41	0.41	5.37	11.43
UV_{254} absorbance (cm^{-1}) [c]	0.01	0.07	0.00	0.24	0.25
Alkalinity (mg $CaCO_3$/L)[d]	184	76.5	56	62	175
Conductivity (µS/cm)[e]	228	390	211	169	749
Turbidity (NTU)[f]	1.23	0.47	0.10	0.21	0.39
Zinc (µg/L)[g]	4.66	14.6	87.7	19.0	76.0
Titanium (µg/L)	4.44	20.6	65.6	4.44	8.87
Silver (µg/L)	0.89	2.94	15.79	5.49	0.66
Sodium (mg/L)	66.1	31.1	11.9	18.4	112
Calcium (mg/L)	0.16	23.9	6.24	7.68	49.3
Potassium	2.35	2.64	1.42	1.53	24.1
Magnesium (mg/L)	0.08	8.73	4.41	6.52	15.9
Chloride (mg/L)	4.95	89.6	9.70	2.44	212
Nitrate	0.35	8.93	0.56	0.40	28.4
Sulfate	16.8	19.6	42.1	34.6	49.3

[a]pH measured with AR20 pH/conductivity meter (Fisher Scientific).

[b]Total organic carbon measured with Shimadzu TOC-V instrument (Shimadzu Scientific Instruments).

[c]Absorbance at 254 nm measured with DR/4000 Spectrophotometer (HACH).

[d]Alkalinity measured by titrating to pH 4.6 with 0.1N HCl.

[e]Conductivity measured using SensION 5 conductivity meter (HACH).

[f]Turbidity measured using 2100N turbidimeter (HACH).

[g] Elemental metal analyses were conducted by ICP-MS (Agilent).

Table S4. Range of metals detected in finished drinking water samples.

Finished water sample	Ag NPs (μg/L)	TiO_2 NPs (μg/L)	ZnO NPs (μg/L)
Coagulation/ Flocculation/ Sedimentation (CFS) -Groundwater (GW)	50-105	28-243	548-1152
CFS - Surface Water (SW)	62-305	91-237	344-1287
CFS - Synthetic freshwater (SFW)	20-155	76-297	688-2165
CFS - Synthetic freshwater with NOM (SFW_NOM)	5-107	14-465	1131-2196
CFS - Wastewater effluent (WWeff)	0-201	0-72	515-3200
Microfiltration (MF) - GW	143-743	516-1026	453-634
MF – SW	21-227	266-338	438-2261
MF – SFW	0-55	3-17	1197-1928
MF - SFW_NOM	41-163	272-1330	689-1004
MF – Wweff	0-52	33-283	203-1904
Ultrafiltration (UF) - GW	0-24	7-15	0-55
UF – SW	0-44	0-8	588-3202
UF – SFW	0-30	6-158	1215-2004
UF - SFW_NOM	0-7	0-43	415-995
UF – Wweff	0-19	0-5	0-887

Table S5. Comparison of NP removal (%) by conventional and advanced treatment determined by mass by ICP-MS (n=3 experiments for each NP in each water type ± standard deviation). For each NP and water type, the most effective treatment is bolded. If all treatments were equally effective, no treatments are bolded.

NP and water type	Conventional Removal (%)	Microfiltration Removal (%)	Ultrafiltration Removal (%)
Ag - Groundwater (GW)	90.7 ± 6.38	53.8 ± 32.7	**99.6 ± 2.65**
Ag - Surface Water (SW)	79.6 ± 12.8	85.5 ± 10.4	**98.2 ± 2.44**
Ag - Synthetic Freshwater (SFW)	97.9 ± 1.19	95.6 ± 6.48	98.6 ± 4.68
Ag - Synthetic Freshwater with NOM (SFW_NOM)	87.5 ± 17.7	92.1 ± 4.23	**99.7 ± 0.37**
Ag - Tertiary Wastewater Effluent (WWeff)	96.7 ± 6.96	98.8 ± 1.35	99.7 ± 0.62
TiO_2 – GW	95.3 ± 3.57	64.0 ± 6.23	**99.5 ± 0.14**
TiO_2 – SW	91.5 ± 3.86	83.1 ± 0.81	**99.8 ± 0.24**
TiO_2 – SFW	94.1 ± 3.20	**99.6 ± 0.21**	95.6 ± 0.99
TiO_2 - SFW_NOM	96.6 ± 0.96	56.2 ± 25.8	**98.7 ± 1.58**
TiO_2 – WWeff	96.3 ± 4.14	89.1 ± 2.08	**100 ± 5.09**
ZnO – GW	51.6 ± 7.92	50.9 ± 6.29	**98.2 ± 3.81**
ZnO – SW	51.7 ± 7.12	31.4 ± 24.1	3.93 ± 33.0
ZnO – SFW	4.49 ± 2.42	17.3 ± 5.62	15.0 ± 7.20
ZnO - SFW_NOM	0.46 ± 2.42	44.4 ± 26.1	64.0 ± 16.2
ZnO – WWeff	39.5 ± 23.6	62.8 ± 40.6	64.1 ± 22.8

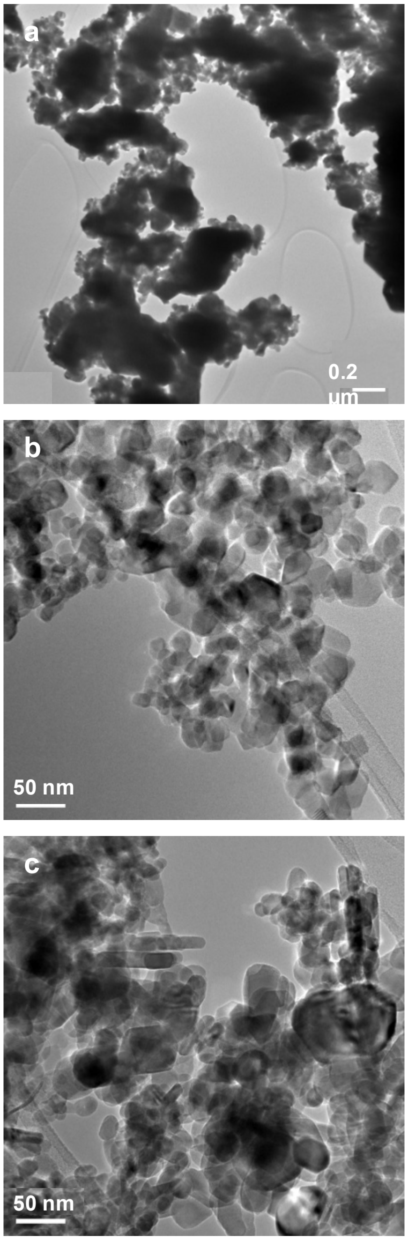

Figure S1. Transmission electron micrographs of a) silver, b) TiO_2, and c) ZnO NPs in ultra-pure water. Note the difference in scale between the images.

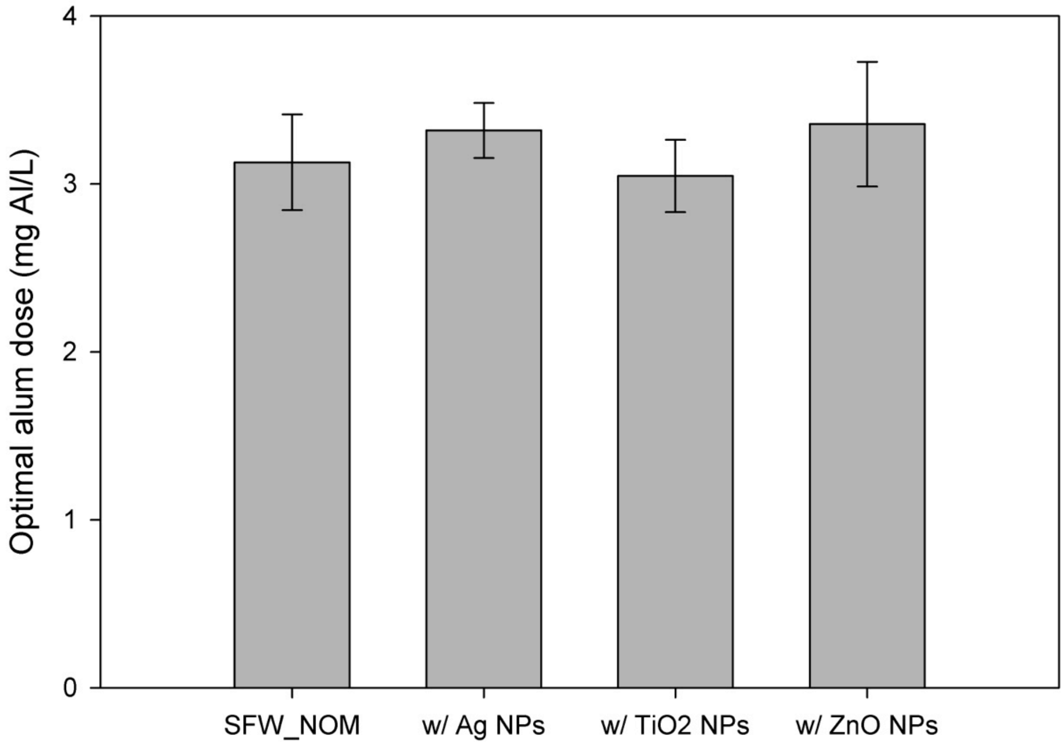

Figure S2. Optimal alum dose for turbidity removal from synthetic freshwater with NOM with and without 1mg/L spiked Ag, TiO_2, and ZnO NPs (n=3 for each NP in each water as well as SFW_NOM without any NPs, average and standard deviation).

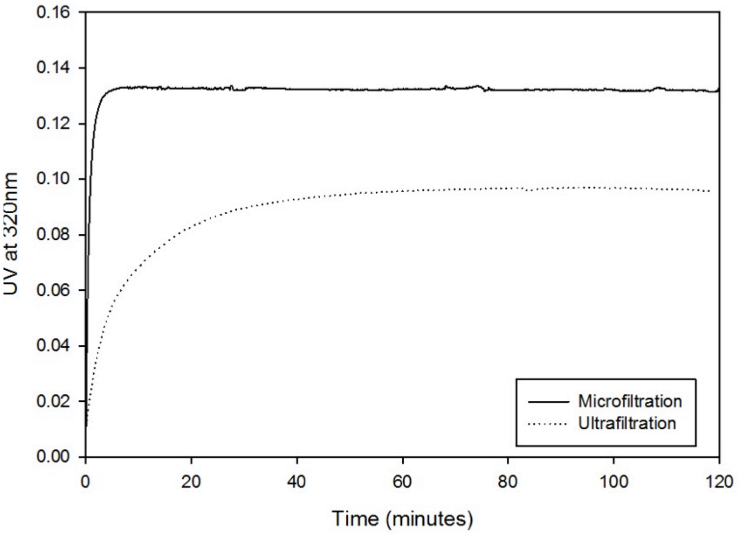

Figure S3. Sample curve of UV absorbance of 1 mg/L TiO_2 NPs in SFW_NOM following micro- and ultra- membrane filtration. UV absorbance at 320nm was monitored for two hours of filtration. All of the UV absorbance from all experimental conditions looked similar to this sample graph.
